# Accurate Multilevel Classification for Wildlife Images

**DOI:** 10.1155/2021/6690590

**Published:** 2021-04-01

**Authors:** Francisco Gomez-Donoso, Félix Escalona, Ferran Pérez-Esteve, Miguel Cazorla

**Affiliations:** Institute for Computer Research, University of Alicante, P.O. Box 99. 03080 Alicante, Spain

## Abstract

The most common approaches for classification rely on the inference of a specific class. However, every category could be naturally organized within a taxonomic tree, from the most general concept to the specific element, and that is how human knowledge works. This representation avoids the necessity of learning roughly the same features for a range of very similar categories, and it is easier to understand and work with and provides a classification for each abstraction level. In this paper, we carry out an exhaustive study of different methods to perform multilevel classification applied to the task of classifying wild animals and plant species. Different convolutional backbones, data setups, and ensembling techniques are explored to find the model which provides the best performance. As our experimentation remarks, in order to achieve the best performance on the datasets that are arranged in a tree-like structure, the classifier must feature an EfficientNetB5 backbone with an input size of 300 × 300 px, followed by a multilevel classifier. In addition, a Multiscale Crop data augmentation process must be carried out. Finally, the accuracy of this setup is a 62% top-1 accuracy and 88% top-5 accuracy. The architecture could benefit for an accuracy boost if it is involved in an ensemble of cascade classifiers, but the computational demand is unbearable for any real application.

## 1. Introduction

The most common pipeline for object recognition relies in the prediction of the most specific class as stated by the dataset that is used for training the system. For instance, a dataset could be composed of the categories “pedestrian,” “bike,” “car,” “van,” and “motorbike.” A classic machine learning approach using a labeled dataset would state if the input sample match any of the classes. Nonetheless, every category is inherently organized as a taxonomic tree. For instance, “bike” and “motorbike” are “two-wheel vehicles”; “car” and “van” are “four-wheel vehicles”; likewise, “two-wheel vehicles” and “two-wheel vehicles” are “vehicles,” and “vehicles” and “pedestrian” are “urban objects.” Thus, we organized plain categories in a taxonomic tree. The categories that are grouped under the same node share common features.

Tackling the classification problem within this framework introduces several advantages. First, the features that would learn the classifier are grouped, so it does not have to learn roughly the same features for several, slightly different categories. Then, in this case, the classifier states the proper category for each level of the taxonomic tree, so it would provide a set of predictions at different abstraction levels.

Thus, in this paper, we study different architectures, approaches, and data setups focused on performing classification on a multilevel fashion. To do so, we tackled the iNaturalist challenge, as it sets a multilevel classification problem, and being a challenge as it is, it also provides an easy comparison framework. In addition, being able to automatically recognize wildlife entities could be applied for a range of different applications. For instance, it could be used for early detection of plagues, to easily analyze the migration habits of several animals, or for protecting endangered flora and fauna population. So far, the precise identification of species is required to be performed by an expert. This takes time and effort. Thus, being able to automatically state the species of a sample could lead to take early actions that would dramatically reduce the consequences of, as we mentioned before, an insect plague in agricultural plantings.

Specifically, the main contributions of this work include the following:

- A study of different convolutional backbones and setups to perform multilevel classification
- A study of different methods to create ensemble models for improving the accuracy of the whole system
- Application of the approach to tackle the iNaturalist challenge for automatically recognizing wildlife entities in images

The rest of the document is structured as follows. First, we briefly discuss related works to classic and multilevel classification approaches and wildlife entity automatic recognition in Section 2. In Section 3, a summary of the details regarding the iNaturalist dataset are given, which is the dataset we use in our experiments. Then, in Section 4, we explain the architectures that were involved in this study, the ensemble models, and the data setup we used in the experiments. The results of the exhaustive experimentation we carried out are discussed in Section 5. Finally, the conclusions of the work and future research directions are drawn in Section 6.

## 2. Related Works

### 2.1. Image Classification

Multiclass classification of images has been a widely studied topic in the history of artificial intelligence. One of the simplest approaches to performing multiclass classification is to train a series of binary classifiers, where the output of each classifier is used to produce the final multiclass classification. This approach was explored in early neural networks and in support vector machines. The solution, although it is simple, also has serious drawbacks. For example, the feature space is not properly scanned and can lead to overfitting issues. In the past, a single classifier that made multiclass predictions was theoretically introduced. However, it could not be tested due to lack of computing power. With the emergence of massively parallel platforms such as GPUs and the deep learning paradigm, this approach has been widely explored. In [1–3], the traditional approach of a binary classifier set is compared to a pure multiclass classifier. The authors claim that the multiclass approach has several advantages, such as reduced training and inference time and a broader exploration of the feature space. However, these approaches do not take advantage of the fact that labels are organized taxonomically. In [4], it is concluded that multiclass classification works best with well-balanced data, while the approach that uses a set of classifiers works best in the presence of unbalanced data.

Since the recent increase in popularity of neural networks, the research of new network architectures has experimented a great development. The increasing number of layers in modern networks amplifies the differences between architectures and motivates the exploration of different connectivity patterns. One of the most popular architectures was the ResNet, and this kind of neural network achieved an impressive performance on many challenging image recognition tasks, such as ImageNet. However, in [5] a comparison is made with other modern architectures. Unlike ResNet architectures, DenseNet concatenates feature maps learned by different layers which increases variation in the input of subsequent layers and improves efficiency. The authors assure that compared to Inception [6] networks, which also concatenate features from different layers, DenseNet are simpler and more efficient. There are other interesting architectures that have recently emerged, one of them is EfficientNet network, and in [7], it is said that in general, the EfficientNet models achieve both higher accuracy and better efficiency over existing convolutional neural networks (CNNs), reducing parameter size, and floating point operations per second (FLOPS). Compared with the widely used ResNet50, EfficientNetB4 uses similar FLOPS and improves significantly the results on ImageNet.

### 2.2. Wildlife Identification

In 2017, Brust et al. [8] trained an object detection method YOLO to extract cropped images of gorilla faces. Once the faces are extracted, they trained a CNN model that had an accuracy of 90%. In this work, the authors discuss how deep learning could be really helpful for ecological studies, specifically, in the fields of identification of natural species, spatiotemporal coverage, and socioecological insights. Another significant work is [9], where the authors take the iNaturalist 2017 dataset as a basis to analyze the existing difficulties in identifying plants and animals. Wildlife identification belongs to a specific field of image classification in which the different categories have great visual similarities between them. This kind of identification is usually called fine-grained classification. For instance, facial identification can be seen as a type of classification with similar visual features. However, because of the underlying geometric similarity between faces, current approaches tend to perform a large amount of specific face preprocessing [10–12]. Moreover, images of natural species have their own characteristics, and for instance, individuals from the same species can differ in appearance due to sex and age and may also appear in different environments. In [13], a solution for the identification of wild animals is discussed, and in this case, the work focuses on identifying animals among 20 species from low-quality images taken from camera traps. The authors claim that unbalanced data are one of the biggest drawbacks to building an effective solution. They also pay attention to other facts, such as data augmentation or using modern network architectures as EfficientNet. However, although they use ensemble learning and admit a great improvement on these systems, it does not go too deep into them.

Due to the similarity between the different categories, only a few experts can identify accurately the corresponding class, so the number of images is usually fewer as detailed expert annotations are more difficult to obtain. To solve this problem, Cui et al. [14] proposed a data transfer learning scheme. However, this technique requires retraining models using large datasets without obtaining significant performance compared to the needed computation time. Furthermore, as we go down into the spectrum of similarity, the number of instances in each class becomes smaller. This motivates the need for automated systems that are able to discriminate between a large number of potentially similar categories, having only a small number of examples for some categories.

### 2.3. Multilevel Classification

Multilevel or hierarchical classification is a very discussed problem within the machine learning community. For instance, in [15], the authors propose a cascade of classifiers to perform Attention Deficit Hyperactivity Disorder from a set of traditional features. This very same approach is used in [16] to create a generic multilevel classifier for medical datasets. Following on, different multilevel architectures are explored in the context of deep learning in [17]. The authors also applied their findings to a Diptera dataset. Finally, a comprehensive and exhaustive review on multilevel and hierarchical classification is provided in [18]. In this review, the multilevel approaches are classified in one classifier per node, which consists on a binary classifier per category; one multilabel classifier per level; and one classifier per parent node, which is also known as cascade of classifiers.

### 2.4. Related Datasets

There are some state-of-the-art datasets that include images of categories with great visual similarities among them. Currently, there are fine-grained datasets related with natural species, for instance, we found datasets about birds [19–21] and dogs [22, 23]. The ImageNet [24] dataset is not usually defined as a fine-grained dataset; however, it does contain several groups of fine-grained classes, including about 60 species of birds and 120 breeds of dogs. Many of these data sets were built with the intention that they would have a uniform distribution of images across the different categories. Another characteristic that this type of datasets usually has is that they only contain images of a single domain, for example, images of birds. However, they do not usually have similar categories from different domains in the same set, such as fungi, plants, insects, and reptiles.

## 3. The iNaturalist Dataset

To train, test, and validate the architectures, we used the iNaturalist 2019 dataset as provided by the corresponding Kaggle challenge (https://www.kaggle.com/c/inaturalist-2019-fgvc6). This dataset is composed of a high number of wildlife samples, which are labeled in a hierarchy fashion following the taxonomic rank of the biological entities. Namely, they provide the category for each level of the taxonomic tree, from first to last level. The dataset depicts images of 1010 different plants, insects, birds, and reptilians.

**Figure 1:**
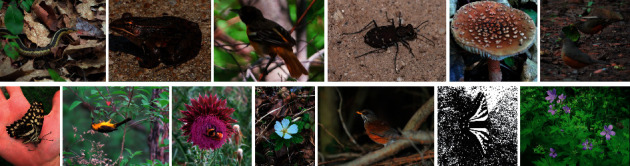
Samples of subjects present in the dataset.

The iNaturalist dataset has a total of 268.243 images, each containing one of the different animal and plant species to classify. All images are labeled with the species to which each individual belongs, and in each case, we have the complete taxonomic tree of the corresponding species, as we mentioned earlier. The shape of this hierarchy is specified is as follows (from higher abstraction to the finest grain category): Kingdom, 3 categories; Phylum, 4 categories; Class, 9 categories; Order, 34 categories; Family, 57 categories; Genus, 72 categories; and Species, 1010 categories. As stated before, each image has only one category assigned for each level. Some random samples of the iNaturalist dataset are shown in Figure 1. Finally, it is worth mentioning that an exhaustive analysis of this dataset could be found in [9].

Despite providing images of fine quality and resolution, and a high number of samples, the dataset has some issues that could affect the performance of the algorithms that are trained with it.

**Figure 2:**
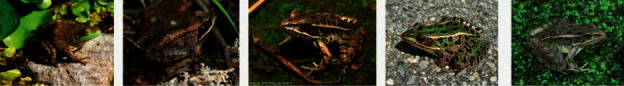
Similarity between species.

For instance, species that share the same taxonomic categories are more similar to each other and, in some cases, can only be distinguished from some small details. For instance, in Figure 2, different species of frogs belonging to the dataset are shown. As it can be seen, distinguishing the species to which each image belongs is a difficult task that can only be done precisely by an expert, due to the high visual similarity between them. The similarity between categories increases as the categories are more fine-grain.

**Figure 3:**
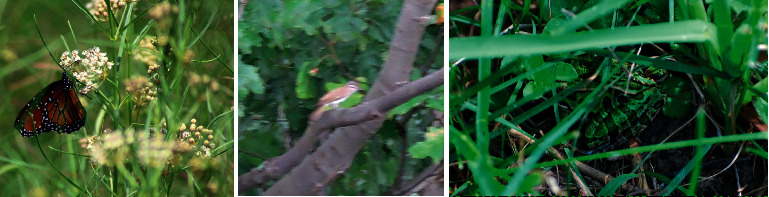
Samples that show challenging conditions for automatic classification algorithms.

Another problem of this dataset that includes flora and fauna at the same time is that elements belonging to different classes appear at the same time in some photographs. In other words, insects or other animals may appear in an image labeled as vegetation. For instance, in the leftmost image of Figure 3, we can clearly observe an insect, but the labeled category is a type of plant. This kind of samples could also be challenging for the algorithms that are trained on this dataset.

In addition to that, in several occasions, the subject to be identified and the background of the image appear blurred or with a very low quality. An example of this can be seen in the central image of Figure 3, where the image has a low resolution and the bird is hard to identify. There are other cases, in which regardless of the quality of the image, the subject to identify is barely perceptible or is quite hidden in the image. In the rightmost image of Figure 3, we can see that an amphibian appears in the photograph, but only part of it is seen, since it is hidden among the grass.

Considering the features discussed here, this dataset is suitable for properly benchmarking any multilevel classification approach, also bearing in mind that some samples are extremely challenging to classify, even for a human, due to the ambiguity they represent.

## 4. Accurate Multilevel Classification

**Figure 4:**
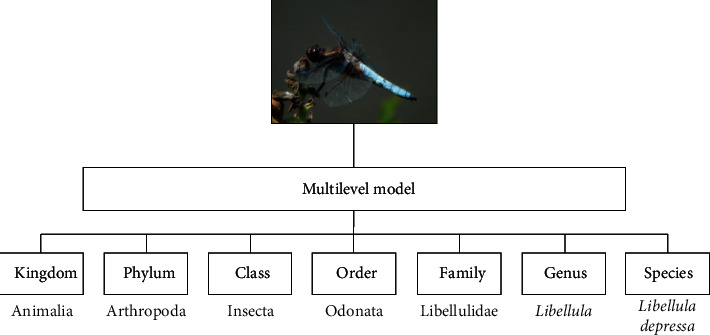
The proposed architectures are able to provide the most probable category for each level in a taxonomic tree.

As explained before, our goal is to provide the best architecture to perform multilevel classification from color images. Namely, given a taxonomic classification tree, the architectures predict the most probable category for each level of the mentioned tree taking a single color image as an input. An example of the prediction provided by our architectures is shown in Figure 4.

To do this, we put to test different deep learning-based convolutional backbones. The architectures of choice were ResNet50 [25], InceptionV3 [26], DenseNet [5], and EfficientNet B5 [7]. These architectures were chosen because they are state of the art, reportedly achieving great accuracy in other datasets such as the ImageNet one. In addition, we also put to test different data setups and ensemble methodologies in order to provide the best configuration to tackle multilevel classification problems.

### 4.1. Classification Convolutional Backbones

As expected, the architectures mentioned before are intended for classification at the finest grain as possible, so we had to modify them in order to enable the multilevel classification. To do so, we removed the last fully connected layer of each architecture and replaced them for seven parallel fully connected layers. Namely, the feature maps provided by the convolutional backbone are forwarded to seven isolated fully connected layers. Each of these layers is a single-level classifier. We put 7 layers to match the iNaturalist annotations that provide seven levels in the taxonomic tree. The number of output neurons of each layer is different as each level has a different number of categories. For instance, the layer for the level “Species” has 1010 neurons whilst the layer for the level “Family” has 57.

It is worth noting that this methodology was applied for the experiments that involve multilevel predictions, such as the experiments explained in Section 5.3.

The experiments shown in Section 5.1 and Section 5.2 were performed following a flat classifier approach in the sake of comparison. Namely, these architecture do not perform multilevel classification, but they provide directly the finest-grain label as possible. To to this, the convolutional backbone is connected to a single fully connected layer with 1010 neurons, matching the number of categories of the “Species” level, which is the most specific level provided by the dataset, as mentioned before. In all cases, the activation function of the output neurons is softmax, which is defined as in equation (1). This function is applied to generate a probability for each class from the logits provided by the convolutional backbone:

(1) $$\begin{array}{c} \sigma {\left(z\right)}_{i}=\frac{e^{z_{i}}}{\sum_{j=1}^{K}e^{z_{j}}}. \end{array}$$

Whether the benchmarked approach was multilevel or flat, all the architectures involved in the experiments were trained following the same setup. First, the dataset already provides a train set and a test set, so we adopted the splits with no modifications. Some experiments involved different data augmentation techniques, which we applied to the training set as explained in Section 4.2. In each training procedure, the data are shuffled so they are fed to the network with a random order. The optimization procedure was carried out by the Adam solver which was initialized with a learning rate of 0.0001. The Adam weight update protocol is shown in equation (2). The error function to minimize was categorical crossentropy. The architectures were initially configured to be trained on a forever loop, and an early stopping criterium was used to halt the training procedure. This criterium consisted of 10 consecutive tests with no significant improvement in the classification accuracy on the test set. A test was performed every 10 training epochs:

(2) $$\begin{array}{c} m_{t}=\beta_{1}m_{t-1}+\left(1-\beta_{1}\right)g_{t},\\ v_{t}=\beta_{2}v_{t-1}+\left(1-\beta_{1}\right)g_{t}^{2},\\ {\hat{v}}_{t}=\max\left({\hat{v}}_{t/1},v_{t}\right),\\ \theta_{t+1}=\theta_{t}-\frac{\eta }{\sqrt{{\hat{v}}_{t}}+\varepsilon }m_{t}. \end{array}$$

### 4.2. Data Setup

Regarding the data, the architectures were trained on the training split and tested with the test split. Both sets are provided by the dataset itself, and we adopted them with no modifications. The original training split is composed of 187770 samples, whilst the test split has 80529 samples. It is worth bearing in mind that the images of the dataset are of different resolution, so they are resized to fit the input size of each architecture. This original data setup was explored in the experiments and is referred as the “No” data augmentation setting.

Some of the experiments we carried out involve different data augmentation techniques. The data augmentation is a common method to artificially create new samples from the existing ones by slightly modifying them. This method is a default procedure when it comes to train machine learning methods and specially to train deep learning models, which is reportedly used to improve its generalization capabilities. We applied data augmentation on the least represented categories in order to match the number of samples of the most represented category of the dataset.

First, the Standard data augmentation technique consists on the application of a range of different operators. Namely, horizontal and vertical flipping, color channel shifting, Gaussian noise addition, brightness and contrast alterations, and Gaussian blur were randomly combined and applied, with random parameters each. The Standard data augmentation is applied to the training data, so an augmented training set of 353500 samples is generated. This set is the same for all the experiments that involved the Standard data augmentation technique.

Then, the Central Crop data augmentation technique consists on generating new samples by extracting the central patch of each image. The center patch covered over the 80% of the original image. As the images of the dataset are of different resolution, the crop is performed before the resize operation. This data augmentation technique is applied together with the Standard setup described before, so when an experiment is setup with the Central Crop method, it also includes the Standard one. We applied this technique because sometimes the input image depicts too much background in addition to the labeled sample. By cropping the central patch, we try to remove the uninteresting background whilst keeping the visual features of the target sample intact.

Finally, the Multiscale Crop data augmentation technique is about extracting the central crop of the image but at different resolution. In this case, three central crops of random size, ranging from 80% to 50% the size of the original sample, were extracted following the same methodology explained before. In this case, the Standard data augmentation method is also applied alongside the Multiscale one. The reason behind the application of this method is that the relative size of the target sample with respect to the whole image is not fixed. Thus, we can find samples where the subject covers the whole image, and others where it is depicted on a small section of it, and the rest is background.

**Figure 5:**
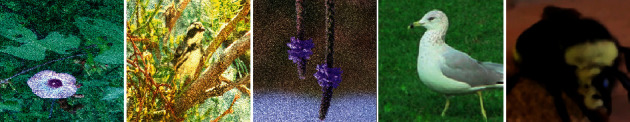
Some random augmented samples.

Some samples after applying these data augmentation techniques are shown in Figure 5.

### 4.3. Ensemble Methodologies

A common technique to improve the results of a classification system is to combine the decision of different classifiers to provide the final result. The goal of this method is to enhance the strong points while complementing the weakest features of each classifier. We implemented three different ensemble models: boosting, stacking, and a cascade of classifiers which is a collection of specialized classifiers. These ensemble methodologies are explored in the experiments shown in Section 5.4.

Boosting [27] is based on weighting the decision of each architecture based on its theoretical accuracy on the validation set as described in equation (3). In our case, different architectures are fed so the individual decisions are computed. Then, the results of each architecture are multiplied by its theoretical accuracy. Next, the results of all the architectures are summed together and the highest scores for each level are returned as the final decision of the ensemble:

(3) $$\begin{array}{c} \hat{d\left(x\right)}=\sum y_{i}\cdot w_{i}. \end{array}$$

Regarding the stacking technique [28], it is based on creating an intelligent system that learns the mistakes that make a range of other classifiers, which outputs are used as the input, in order to correct them. In our case, we trained a fully connected layer that takes the output of a range of different multilevel architectures as an input and predicts the final multilevel decision.

Finally, the specialized cascading classifiers [29] consist of creating specialized classifiers for each possible category in a multilevel approach. Thus, the prediction of a specific level is trusted to be correct in order to forward the data to the proper specific classifier which will provide the prediction for the next level, namely, the next classifier. As we have 9 levels deep and more than 1000 categories, training one classifier for each is unbearable. Thus, we only performed this technique for the Phylum level, which includes 4 different categories. Thus, in our incarnation of this approach, a classifier is in charge of deciding the proper Phylum of the input sample. Then, based on this decision, the sample is forwarded to the classifier of the predicted Phylum so it states the rest of the more specific levels. Namely, we trained a general classifier that predicts the Phylum, and a specialized classifier for each Phylum that predicts the category for the rest of the levels.

## 5. Experimentation and Discussion

In this section, the results of the experiments we carried out are reported. In addition to each setup, we also provide the top-1 and top-5 accuracy.

All the experiments were conducted on the same machine, which features an Intel i7-7700 @ 3.6 GHz CPU with 16 GB of DDR3 RAM. The chipset of choice is Z370. All the computations were accelerated by a Nvidia Quadro P6000 GPU unit. Regarding the software, the SO is Ubuntu 18.04 LTS and all the architectures were implemented on Keras 2.2.4 and tensorflow 1.4.0 frameworks using CUDA 9.0 and CuDNN.

### 5.1. Baseline Convolutional Backbone Flat Classifiers

In this section, the experiments are intended to compare different convolutional backbones. In addition to that, it is also intended to discover whether the data augmentation impacts on the accuracy. It is worth noting that these experiments do not involve multilevel classification, but just flat classification on the most specific level of the taxonomy. The results are reported in Table 1.

As it can be seen, the most accurate convolutional architectures are the DenseNet201 and the EfficientNetB5 with a marginal accuracy difference between them. On one hand, the DenseNet architecture features residual connections between every convolutional block. This fact allows the network to explicitly learn powerful multiscale features that are very helpful for correctly classifying the images in which the sample is depicted in a small part. In a convolutional pipeline with no residual connections, the mentioned visual features would disappear as per effect of the convolution and pooling operations in the earliest stages of the network. On the other hand, the EfficientNet family of architectures was created by performing a Neural Architecture Search. Namely, this collection of architectures was proposed by an intelligent system that aims to achieve the best trade-off between accuracy and runtime. The EfficientNetB5 is the most powerful in terms of accuracy, and it provides the best top-1 and top-5 results too in our experiments.

Regarding the inclusion of data augmentation, it clearly impacted the accuracy of every architecture. As it can be seen, all of them experienced a significant accuracy boost when data augmentation was involved in both top-1 and top-5 metrics.

Thus, we can conclude that the setups involving data augmentation techniques, and DenseNet101 and EfficientNetB5 convolutional backbones provide the best accuracy.

### 5.2. Resolution Experiments on Flat Classifiers

In this section, we put to test the impact of the input resolution on the accuracy of the architectures for flat classification. No multilevel prediction is involved in these experiments. Despite being DenseNet101 and EfficientNetB5, the architectures that provided the best performance on the experiments of Section 5.1, they are very computationally expensive. Namely, due to hardware restrictions, we cannot test these networks with a resolution greater than 300 × 300 pixels. Thus, we adopted ResNet50 to carry out these experiments up to 350 × 350 and we will assume that the results apply to DenseNet and EfficientNetB5.

The results of testing ResNet50 with a range of different resolutions are shown in Table 2. For this particular problem, the accuracy is proportional to the resolution. Namely, the accuracy improves as the resolution of the input images increases. It is expected that the improvement becomes marginal at a certain resolution onwards, but we cannot reach that limit due to hardware limitations as explained before. It is also worth noting that the accuracy of ResNet50 with a resolution of 350 × 350 is similar to the accuracy achieved by EfficientNetB5 with 250 × 250 resolution, being also similarly computationally expensive.

At this point, the setup that provides the best accuracy is DenseNet and EfficientNet with data augmentation and the largest resolution allowed by the hardware, which in our case is 300 × 300 pixels.

### 5.3. Convolutional Multilevel Classifiers

In this section, we benchmarked the best setup for performing multilevel classification. As the experiments we performed before on flat classification conclude, only DenseNet and EfficientNetB5 are considered because they provide the best accuracy. In addition, 250 × 250 and 300 × 300 resolutions are put to test. Finally, the three different types of data augmentation explained in Section 4.2 are also benchmarked.

The results of the experiments on multilevel classification are reported in Tables 3 and 4. Regarding the convolutional backbone, the one that provides the best top-1 and top-5 accuracy regardless the input image resolution and the data augmentation type is EfficientNetB5. EfficientNetB5 consistently outperformed DenseNet201 in every experiment if it is compared pairwise with the same setup.

The impact of the resolution is also noticeable in this experiment, as the increment of resolution led to an accuracy boost in every experiment as well. This is expected as the visual features of the samples are of more quality, and they also can reach deeper in the convolutional pipeline, which improves the classification accuracy. This effect is more noticeable on the DenseNet101 architecture as it explicitly provides multiscale feature extraction through its dense residual connections.

The inclusion of Central Crop and Multiscale Crop data augmentation techniques increased the accuracy of the models too. However, the improvement is marginal respect to the Standard method. It seems that it is preferable to enlarge the input resolution rather than to apply Central Crop or Multiscale Crop data augmentation techniques when possible. In addition, we can conclude that the Standard data augmentation technique indeed helps to improve the generalization capabilities and the overall accuracy of the model in this multilevel classification configuration.

**Figure 6:**
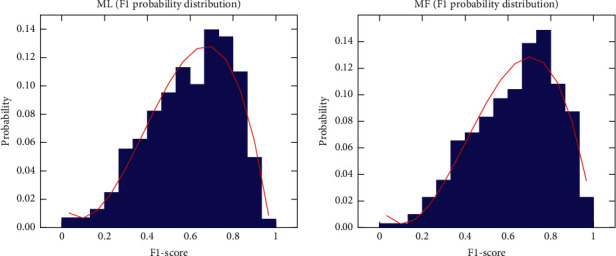
Probability distribution of F1-score for the ML (leftmost) and MF (rightmost) experiments.

As we can see in Figure 6, the probability distributions computed with the F1-score results show that the performance of both models look pretty similar. F1-scores for the ML model can be represented as a normal distribution of 0.60 mean and 0.04 of typical deviation, while the results for MF model can be expressed as a normal distribution of 0.62 mean and 0.03 of typical deviation.

These distributions mean that the ML model has approximately 659 classes (65.2%) whose F1-score is between 0.56 and 0.64, and 964 classes (95.4%) whose F1-score varies from 0.52 to 0.68. On the other hand, the MF model has approximately 659 classes (65.2%) whose F1-score is between 0.59 and 0.65, and 964 classes (95.4%) whose F1-score varies from 0.56 to 0.68.

In the light of the experiments, both models (DenseNet201 and EfficientNetB5 with Multiscale Crop) show a similar performance, DenseNet201 is slightly superior in the F1-score and AUC score test, whilst EfficientNetB5 is moderately superior in the top-1 and top-5 test, which we considerate crucial, so we selected EfficientNetB5 as the convolutional backbone for performing multilevel classification of wildlife imagery, with input images of 300 × 300 pixels resolution and Multiscale Crop data augmentation. However, it is worth mentioning that the improvement over the Standard data augmentation configuration is marginal.

### 5.4. Multilevel Ensemble Approaches

In this section, we put to test the performance on multilevel classification of the ensemble setups discussed on Section 4.3. As the aim of the ensemble models is to combine different approaches so that their weaknesses are compensated and their strong points enhanced, we chose the setups MF and ML, as reported in Table 3, to create the ensembles. These setups provide the best accuracy levels thus far and feature two different convolutional backbones.

The results of both ensemble methods, boosting and stacking, are reported in Table 5. In the light of the results, both methods led to an important improvement in terms of accuracy compared with using just one architecture. Furthermore, the boosting approach provides an even better accuracy, reaching a top-1 71%.

We also tested the cascade of classifiers approach. Despite a pure cascade of classifiers would involve a classifier per category in each level, we only trained specialized classifiers for each Phylum in this case. As we have 9 levels deep and more than 1000 categories, training one classifier for each is unbearable. The setup of choice for the classifiers was based on the experiment ML, as reported in Table 3. The results for this setup show a slightly better accuracy compared with the original ML experiment. Nonetheless, even if the improvement was not marginal, the requirement of a single classifier per category in each level makes this approach very time consuming, which could be inadequate for a range of applications.

**Figure 7:**
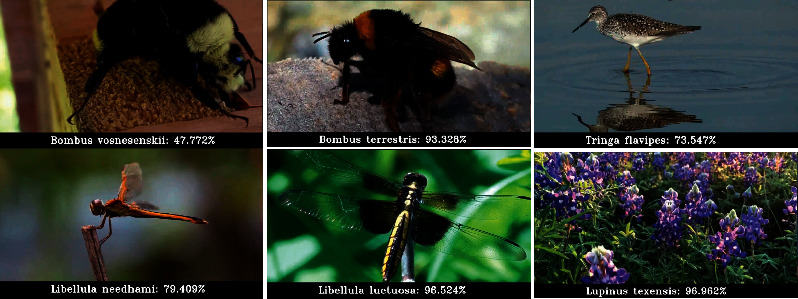
Random samples correctly classified as provided by the setup EC.

Finally, some results of correctly classified subjects as provided by the EC setup are shown in Figure 7. As it can be seen, the approach is able to work with images that do not depict the subject entirely, with cluttered scenarios, with several subjects in the same picture and with a range of different poses.

**Figure 8:**
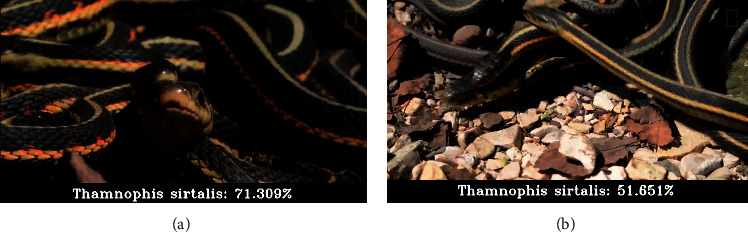
A sample of *Thamnophis sirtalis* (correctly classified) and sample of *Thamnophis elegans* (incorrectly classified). Note the high visual similarity of both species.

In addition, some errors are shown in Figure 8. In this case, the first image depicts a sample of *Thamnophis sirtalis* and the second one a sample of *Thamnophis elegans* incorrectly classified. The main difference between these two species is that the first has a dash-like pattern of red marks along the lateral parts of its body. This kind of error happens sometimes with species that look so similar.

## 6. Conclusion and Future Work

In this work, we have presented an exhaustive study of different methods to perform multilevel classification from color images applied to the problem of classifying wild animals and plant species.

In order to solve this problem, data from different competitions of *iNaturalist* have been fused and processed with data augmentation techniques. Moreover, several different state-of-the-art architectures have been adapted to the taxonomy of our problem to find the best model.

Our experiments show that increasing the resolution of the images impact on the final accuracy, as the finer details are very important to determine the exact specie of each being are preserved. In addition to that, the best architecture is EfficientNetB5 with the Multiscale data augmentation method. Furthermore, the ensemble models show even better accuracy, being the boosting technique that provides the best results.

It is important to note that, additionally to the results presented in this paper, our system has been applied with success to a range of real-life videos that contain moving species. A sample of this functionality could be seen at https://youtu.be/rzEIYt4Gj0A. This test has let us check the performance of our system in real scenarios, being capable of classifying more than 1000 species with high robustness.

Regarding the limitation of the methods, they still have room for improvement in terms of accuracy. In addition to that, it is worth noting that the error of the classifiers is greater as they got deeper in the taxonomy. Namely, the classifier of the Species level is the most prone to error. This is expected as the samples of different categories are more visually alike. Thus, in future research studies, we plan to tackle this problem so that the accuracy is kept no matter the depth level in the taxonomic tree.

## Figures and Tables

**Table 1:** Results on flat classification for different convolutional backbones and data augmentation.

ID	Conv. backbone	Resolution	Data augmentation	Top-1	Top-5
BA	ResNet50	250 × 250	No	0.27	0.62
BB	ResNet50	250 × 250	Standard	0.45	0.73
BC	InceptionV3	250 × 250	No	0.34	0.63
BD	InceptionV3	250 × 250	Standard	0.46	0.75
BE	DenseNet201	250 × 250	No	0.33	0.64
BF	DenseNet201	250 × 250	Standard	0.51	0.77
BG	EfficientNetB5	250 × 250	No	0.36	0.65
BH	EfficientNetB5	250 × 250	Standard	0.53	0.8

**Table 2:** Results of flat classification for a range of different input image resolutions.

ID	Conv. backbone	Resolution	Data augmentation	Top-1	Top-5
RA	ResNet50	200 × 200	Standard	0.43	0.72
RB	ResNet50	250 × 250	Standard	0.45	0.73
RC	ResNet50	300 × 300	Standard	0.48	0.76
RD	ResNet50	350 × 350	Standard	0.53	0.83

**Table 3:** Results for multilevel classification that includes different convolutional backbones, input image resolution, and data augmentation techniques.

ID	Conv. backbone	Resolution	Data augmentation	Top-1	Top-5
MA	DenseNet201	250 × 250	Standard	0.51	0.78
MB	DenseNet201	250 × 250	Central Crop	0.52	0.79
MC	DenseNet201	250 × 250	Multiscale Crop	0.51	0.78
MD	DenseNet201	300 × 300	Standard	0.58	0.83
ME	DenseNet201	300 × 300	Central Crop	0.59	0.84
MF	DenseNet201	300 × 300	Multiscale Crop	0.61	0.85
MG	EfficientNetB5	250 × 250	Standard	0.53	0.82
MH	EfficientNetB5	250 × 250	Central Crop	0.53	0.81
MI	EfficientNetB5	250 × 250	Multiscale Crop	0.54	0.84
MJ	EfficientNetB5	300 × 300	Standard	0.61	0.87
MK	EfficientNetB5	300 × 300	Central Crop	0.62	0.87
ML	EfficientNetB5	300 × 300	Multiscale Crop	0.62	0.88

**Table 4:** Results for multilevel classification that includes area under the ROC curve and weighted F1-score.

ID	Conv. backbone	Resolution	Data augmentation	AUC	F1-score
MF	DenseNet201	300 × 300	Multiscale Crop	0.812	0.62
ML	EfficientNetB5	300 × 300	Multiscale Crop	0.806	0.60

**Table 5:** Results for multilevel ensemble methods.

ID	Setup	Ensemble Mode	Top-1	Top-5
EA	MF + ML	Boosting	0.71	0.95
EB	MF + ML	Stacking	0.68	0.93
EC	ML	Cascade classifiers	0.63	0.89

## Data Availability

The data used to support the findings of this study are currently under embargo while the research findings are commercialized. Requests for data, (6/12 months) after publication of this article, will be considered by the corresponding author.
